# *Culex quinquefasciatus* larvae development arrested when fed on *Neochloris aquatica*

**DOI:** 10.1371/journal.pntd.0009988

**Published:** 2021-12-03

**Authors:** M. Florencia Gil, Marisol Fassolari, Marina E. Battaglia, Corina M. Berón

**Affiliations:** Instituto de Investigaciones en Biodiversidad y Biotecnología (INBIOTEC—CONICET); Fundación para Investigaciones Biológicas Aplicadas (FIBA), Mar del Plata, Argentina; Faculty of Science, Mahidol University, THAILAND

## Abstract

*Culex quinquefasciatus* is a cosmopolitan species widely distributed in the tropical and subtropical areas of the world. Due to its long history of close association with humans, the transmission of arboviruses and parasites have an important role in veterinary and public health. Adult females feed mainly on birds although they can also feed on humans and other mammals. On the other hand, larvae are able to feed on a great diversity of microorganisms, including microalgae, present in natural or artificial breeding sites with a high organic load. These two particularities, mentioned above, are some of the reasons why this mosquito is so successful in the environment. In this work, we report the identification of a microalga found during field sampling in artificial breeding sites, in a group of discarded tires with accumulated rainwater. Surprisingly, only one of them had a bright green culture without mosquito larvae while the other surrounding tires contained a large number of mosquito larvae. We isolated and identified this microorganism as *Neochloris aquatica*, and it was evaluated as a potential biological control agent against *Cx*. *quinquefasciatus*. The oviposition site preference in the presence of the alga by gravid females, and the effects on larval development were analyzed. Additionally, microalga effect on *Cx*. *quinquefasciatus* wild type, naturally infected with the endosymbiotic bacterium *Wolbachia* (*w*^+^) and *Wolbachia* free (*w*^−^) laboratory lines was explored. According to our results, even though it is chosen by gravid females to lay their eggs, the microalga had a negative effect on the development of larvae from both populations. Additionally, when the larvae were fed with a culture of alga supplemented with balanced fish food used as control diet, they were not able to reverse its effect, and were unable to complete development until adulthood. Here, *N*. *aquatica* is described as a biological agent, and as a potential source of bioactive compounds for the control of mosquito populations important in veterinary and human health.

## Author summary

*Culex quinquefasciatus*, known as a southern house mosquito, is a domestic and cosmopolitan species widely distributed in the tropical and subtropical regions of the Americas, Asia, Africa, and Oceania. It is strongly associated with humans and other vertebrates, and it has been given a relevant role in the transmission of arboviruses and parasitic diseases, some of them very important in veterinary and human health. Adult females feed mainly on birds, although they can also feed on humans and other mammals, being effective not only in surviving in the environment, but in vectoring pathogens as well. In addition, *Culex pipiens* and *Cx*. *quinquefasciatus*, members of the *Cx*. *pipiens* complex, coexist in a distribution hybrid zone and their mating produces viable offspring, expanding its distribution even more. Moreover, larvae can be developed in different environments, including standing water generated by humans and livestock, being able to exploit food sources found in them. This ability to get adapted to different conditions make it a successful host with great potential to initiate and facilitate the transmission of pathogens, therefore it is essential to develop environmentally friendly control systems that can be used in integrated vector management programs. In this context, the use of microorganisms, like microalgae, with the capability to alter or slow down the development of insects such as *Cx*. *quinquefasciatus* must be exhaustively explored.

## Introduction

The southern house mosquito, *Culex quinquefasciatus*, is a cosmopolitan species widely distributed in the tropical and subtropical areas of the Americas, Asia, Africa, and Oceania. It is well known as a domestic mosquito, developing in rural, semi-urban and urban areas, and having a great success in invading new environments [1,2]. Due to its long history of close association with humans, it has been given an important role in the transmission of arboviruses [3]. It has been described as vector of lymphatic filariasis, West Nile virus, Saint Louis encephalitis virus [4], and as the major vector of Japanese encephalitis virus (JEV) in South-East Asia [5]. It has also been recognized as a competent vector of several arboviruses like Rift Valley fever virus and others, and some protozoa like *Plasmodium relictum* that causes bird malaria [6]. Besides, it is important in vectorizing the dog heartworm *Dirofilaria immitis* [7], chikungunya virus in laboratory conditions [6] and may be involved in Zika virus (ZIKV) urban transmission in Brazil [8]. Adult females feed mainly on birds, although they can also feed on humans and other mammals [2], and besides larvae are able to feed on a great diversity of microorganisms present in natural or artificial breeding sites with a high organic load, being the reason why this mosquito is so successful.

Some reports indicate that the diet of mosquitoes directly influences aspects of growth such as development, longevity, and competition for the transmission of pathogens. In most species, the oviposition site selection is not random and depended on the proportion of organic food sources, the larval density, and food limitation. Additionally, it is associated with larval and adult body sizes, survival until adulthood and number of eggs per raft, as well [9,10]. This is not the same for all mosquito species, so that, for example for *Cx*. *quinquefasciatus* breeding sites, habitats containing sewage water where there are conspecific egg rafts enhanced oviposition levels [11]. As well it has been described that no matter what their larval diet was, adult females were able to live longer when fed on amino acids in their adult diet [12].

A group of frequent microorganisms in mosquito breeding sites with high organic load are microalgae, capable of producing bioactive metabolites, such as allelochemicals, which can confer competitive advantages, such as effects on survival, reproduction or inhibition of the growth of competing species in aquatic ecosystems. For that, microalgae are considered a source of new antimicrobial agents and potential bioherbicide and biological control agents [13]. For example, species such as *Amphora coffeaeformis* or *Scenedesmus obliquus* have been toxic against *Cx*. *pipiens* larvae when were ingested in significant quantities, and the lethal effect could be by metabolites like polyphenolic and unsaturated fatty acids. Some of these compounds have been purified and used against some mosquito larvae, detecting that some of them could have larvicidal activity or that they could produce alterations in their development [14,15]. On the other hand, the microbiota present in mosquitoes, throughout their development, can affect their immunological status and their ability to respond to the action of pathogenic microorganisms [16,17]. In particular, the presence of the symbiont bacteria *Wolbachia* in some insects, including mosquitoes, promotes the induction of reactive oxygen species (ROS) which regulates the activation of immune genes to secrete proteins [18]. Additionally, *Wolbachia* is associated with hematophagous insects being responsible for modulating host metabolism including glycogen pathway, immune pathway activation and supplying micronutrients like B vitamins [19–21].

Species of the *Cx*. *pipiens* complex were found breeding in small artificial containers, like automobile and truck tires, maintained outdoors in travel services and tire-repair stations for long periods of time [22]. During the mosquito sampling activities in this type of artificial breeding sites differences in the development of populations of immature mosquitoes were found, in Buenos Aires province, Argentina. In a group of discarded tires with accumulated rainwater, only one of them had a bright green culture without mosquito larvae, while the other surrounding tires contained a large number of them. The objective of this work was to identify the microalga found as the main microorganism present in this sample, and to analyze its effect on larval development or toxic action against *Cx*. *quinquefasciatus*, and the selection as an oviposition site by its gravid females. Additionally, microalga effect on *Cx*. *quinquefasciatus* wild type, naturally infected with the endosymbiotic bacterium *Wolbachia* (*w*^+^) and *Wolbachia* free (*w*^−^) laboratory lines was explored.

## Methods

### Ethics statement

The protocol for mosquito laboratory rearing, as well for the blood feeding mosquitoes on mice, was reviewed and approved by the CONICET Safe Procedure Policy (NPS: NBTC017-version 1.3) and by Animal Experimental Committee at the Faculty of Exact and Natural Sciences, National University of Mar del Plata (Institutional Committee on Care and Use of Experimental Animals (CICUAL) N° 2555-04-14, OCA 954/19, and Dean’s Office resolution N° 623). The mice were handled in strict accordance with the guidelines of the National Food Safety and Quality Service (SENASA, Argentina) and with the 2011 revised form of The Guide for the Care and Use of Laboratory Animals published by the U.S. National Institutes of Health.

### Microalga isolation and culture conditions

During mosquito sampling activities in artificial breeding sites, in summer 2019, in General Pirán (37°16’37.0"S 57°45’55.0"W), Buenos Aires province, water accumulated in a discarded tire was collected. The principal microorganism present in this sample was a microalga, which was isolated through serial dilutions and cultured in BG-11_0_ agar plates [23] supplemented with 6 mM NaNO_3_ (BG-11). Axenic culture was established after selecting the specific antibiotic (S1 Table) in BG-11_0_ agar plates, allowing the total elimination of the accompanying bacterial flora. Stock cultures were maintained aerobically in 250 mL Erlenmeyer flasks with constant orbital shaking (120 rpm) at 29 ± 1°C under constant light (70 μmol photons m^-2^s^-1^) in BG-11 medium supplemented with neomycin (30 μm/mL). Primary stock cultures were sub-cultured every 3 weeks in BG-11 agar plates, as well as in LB and YPD agar to evaluate the presence of bacteria and fungi, respectively.

To carry out the different assays against mosquitoes, microalga was cultured in sterilized 250-mL transparent glass bottle, air-bubbled (from the bottom), under constant light (70 μmol photons m^-2^s^-1^) at 29 ± 1°C in BG-11 medium supplemented with neomycin (30 μg/mL). Cultures were grown for four days, centrifuged at 1,912 *x g* for 10 minutes and washed with sterile distilled water three times. The supernatant was discarded, and the pellet was resuspended in sterile dechlorinated water to OD_750_ 0.8.

### Microalga characterization

Microalga molecular characterization was performed by PCR amplification of the ribosomal RNA region ITS1-5.8S-ITS2 according to Timmins *et al*. [24] from genomic DNA extracted followed Do Nascimiento *et al*. [25] protocol. PCR amplifications products were analyzed by agarose gel electrophoresis (1%) and sent to an external service for its sequencing (Macrogen Inc., South Korea). DNA sequence data sets were analyzed by BLAST tools [26]. For the phylogenetic analyses, additional ITS1-5.8S-ITS2 coding-sequences of microalgae species and outgroup taxa were obtained from the National Center for Biotechnology Information (NCBI) (http://www.ncbi.nlm.nih.gov). Retrieved sequences were aligned using ClustalW [27]. Dendrogram was constructed with maximum likelihood method and 500 bootstrap using MEGA version 10.1.7 [28].

As during the isolation of the alga, different morphotypes were detected according to the culture conditions, and the objective was to recreate the shape detected in the field samples. For that, the microalga was incubated under different light conditions and constant temperature according to Přibyl [29]. Briefly, overnight culture (OD_750_ 0.4) was divided in three equal parts, two of them were grown under continuous light, while the other one was cultivated in photoperiod condition (16:8 h light: dark cycles). After three days were covered with aluminum foil and incubated for a further 16 h, while a control culture was maintained under continuous light. Microalga cells were collected from each culture and observed under microscope (Nikon E600) using a 100x oil immersion objective.

### Mosquito rearing

In order to analyze microalga effect on *Cx*. *quinquefasciatus* wild type, naturally infected with the endosymbiotic bacterium *Wolbachia* (*w*^+^) and *Wolbachia* free (*w*^−^) treated with tetracycline, laboratory lines were used. Mosquitoes were maintained in the insectary of the Biological Control Laboratory of the INBIOTEC-CONICET, FIBA (Argentina) at standard conditions of 24°C and 80 ± 5% relative humidity (RH), photoperiod (12:12 h light: dark cycles) and fed with commercial fish food (Shulet Carassius). Adult stages were provided with 10% (w/v) sucrose solution and the gravid females were allowed to feed blood from mice for three hours, in a maximum of 10 females per mice, when it was needed.

### Impact of microalga on oviposition substratum preferences by *Cx*. *quinquefasciatus* females

In order to determine if the alga releases some repellent compound, gravid females from *Cx*. *quinquefasciatus* (*w*^*+*^) and (*w*^*-*^) lines were used for oviposition preference analysis. Three days after blood meal, thirty gravid females were individually placed into standard rearing cages (20 x 20 x 20 cm) with two different substrates available for oviposition in transparent cups (6 cm in diameter, 4 cm high). One of them with 20 mL of sterile dechlorinated water, and the other one with 20 mL of alga suspension. Cups were randomly placed into each cage, and the cages were placed randomly on racks in a room with the same environmental conditions as in the insectary [30]. The oviposition site selection was daily recorded for a maximum of 5 days, until the female laid a raft of eggs.

To analyze if microalga suspension is a favorable environment for eggs hatching and larvae development on both *Cx*. *quinquefasciatus* isolines, eggs laid in the oviposition assay were monitored until hatching, and survival of neonate larvae were daily recorded until all died. Neonate larvae hatched in dechlorinated water were considered as a control assay and were daily recorded until molting.

### Effect of microalga on mosquito development and survival

To determine if the microalga was toxic against *Cx*. *quinquefasciatus* (*w*^+^) or the effect was related to nutritional requirements, 50 second-instar larvae (divided in groups of 10 per cup) were placed in transparent cups (6 cm in diameter, 4 cm high) with 20 mL microalga suspension, microalga suspension plus control diet (20 mg of Shulet Carassius fish food every other day), or dechlorinated water with fish food as negative control. The development rate and survival were measured during mosquito development until adult emergence. Additionally, to analyze whether the presence of the endosymbiont bacterium *Wolbachia* modified the effect produced by the microalga, the same treatments were repeated on the *Wolbachia* free isoline (*w*^-^). Larvae were observed daily until adult emergence, and dead larvae and exuviae were removed. At days 3, 7 and 11, larvae of each cup were placed in microscope glass slide and images were captured using a digital camera Olympus DP72 coupled to a Nikon SMZ800 stereoscope, using cellSens Entry imaging software. The total length of the larvae was measured with ImageJ v1.52a software [27] using internal scale from each image. The bioassay was repeated three times.

### Midgut extract activity on the cellular integrity of *Neochloris aquatica*

In order to evaluate if mosquito larvae could digest *N*. *aquatica* cells, 20 midguts from *Cx*. *quinquefasciatus* isolines *w*^+^ and *w*^-^ larvae were dissected. Previously, larvae were externally thoroughly cleaned with distilled water and immobilized on a cold plate. Midguts were dissected in a glass slide placed under Nikon SMZ800 stereoscope with a phosphate buffered saline (PBS) 1X drop. Midguts from each isoline were collected in a pre-chilled 1.5 mL tube containing 100 μL of 50 mM Tris-HCl buffer pH 8, manually homogenized and then centrifuged 10 min at 16,200 *x g*. Supernatant obtained (midgut extract) was transferred to new tubes. Simultaneously, microalga culture was prepared as previously described and concentrated up to 3X.

To analyze the midgut extract activity against *N*. *aquatica*, different quantities of midgut extracts (0.5, 1, 2, 5 and 10 μL) were mixed with 10 μL of microalga culture, completing to a 20 μL final volume with distilled water. Mixtures were incubated at 37°C for 2 hours or overnight. After incubation, qualitative visualization of *N*. *aquatica* cells integrity by light field microscopy and presence/absence of chlorophyll by fluorescence microscopy (Nikon E600) were performed. At the same time, the cellular growth was analyzed by droplet-plate assay. Briefly, 10 μL aliquots of each microalga:midgut extract activity reaction was placed in a BG-11 plate supplemented with neomycin (30 μg/mL) growing in controlled conditions of light and temperature (70 μmol photons m^-2^s^-1^), 30°C during 24 hours.

### Statistical analysis

Oviposition site selection was analyzed using a binomial test. Survivorship and proportion of life stages among treatments were assessed using Kaplan-Meier survival estimator and 2-Way ANOVA multiple comparisons respectively, with GraphPad Prism 8.0.1 (San Diego, CA, USA, www.graphpad.com). As we considered replication to be a random effect, results of the algal impact on the development of mosquitoes were analyzed using a generalized linear mixed models (GLMMs) [31]. In particular, total length of larvae from all three diets and both isolines (*w*^*+*^ and *w*^-^) were analyzed using the *glmer* function of the ‘lme4’ package [32] with a Gamma distribution with inverse function as link function; variables day, diet, isoline and length were considered as fixed effects. Minimum adequate model was assessed using the *dredge* function of the Multi-Model Inference (MuMIn) package [33] based on model ranking by Akaike Information Criterion (AIC) [34,35]. Best model was selected according to Akaike weight (ω_*i*_), which represents the relative likelihood of a model of a set of models considered [36]. Pairwise Wilcoxon rank sum test was used post-hoc to identify which groups differ from each other. GLMM statistics and graphics were performed using RStudio software version 1.3.1056 [37].

## Results

### Microalga characterization

After serial dilutions in BG-11 agar plates, a microalga and one associated bacterium were isolated from standing water taken from an artificial mosquito breeding site. Bacterium was axenically isolated, identified by the amplification of 16S rRNA gene (with 27F and 1492R primers) as *Pseudomonas* sp., and preliminary toxicity bioassay was conducted with second-instar larvae of each mosquito line and no toxic effect was observed. For that, we decided to analyze if the microalga had any effect on the selection of the oviposition site by the gravid females, or toxic effect against larval stages. As it was not possible to isolate the microalga by serial dilutions, it was necessary to select an appropriate antibiotic to eliminate the associated bacterium (S1 Table).

**Fig 1:**
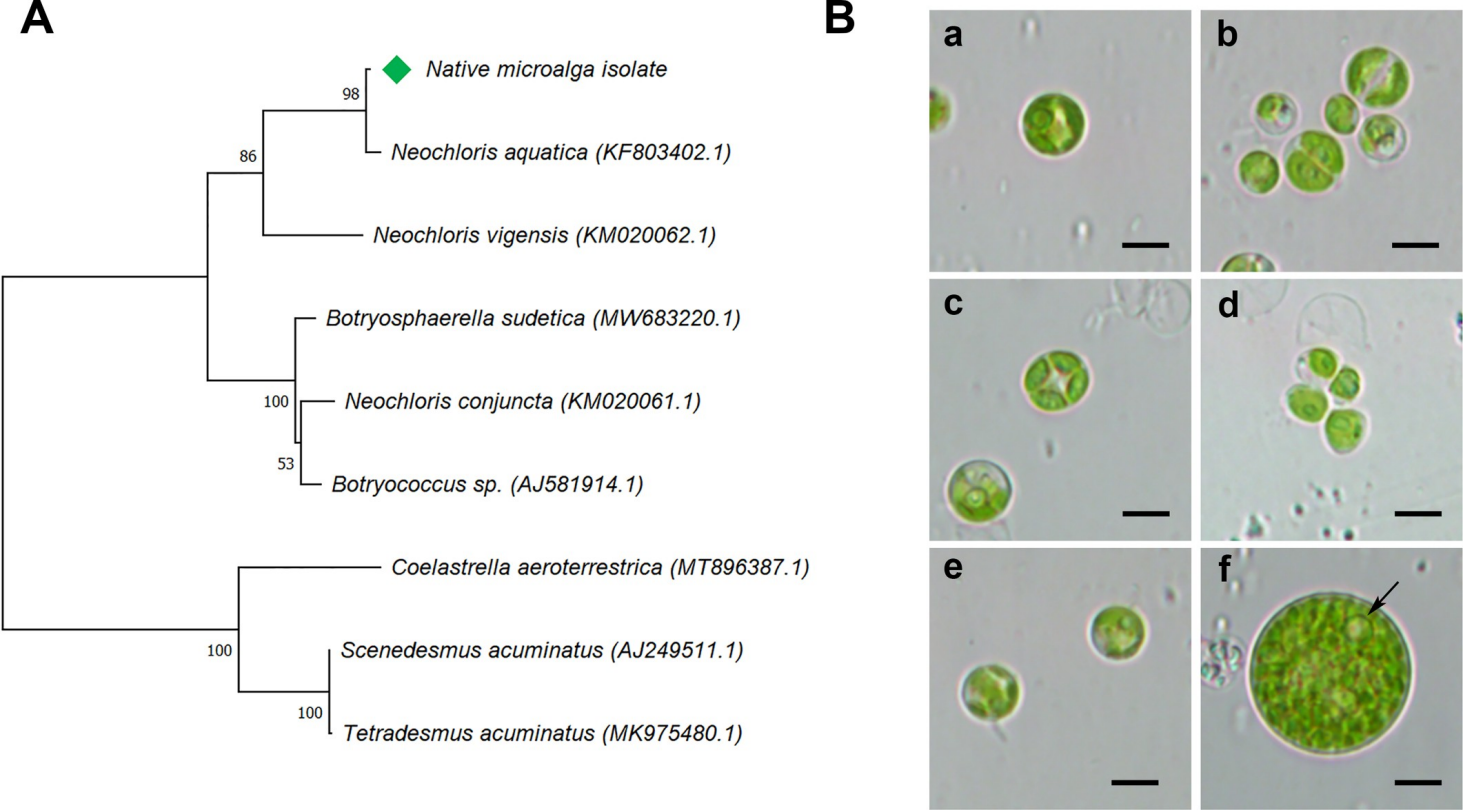
Microalga morphotypes and molecular identification. (**A**) Dendrogram of ITS1-5.8S-ITS2 rRNA region including the native *N*. *aquatica* and the closest related sequences from other microalgae available in NCBI database. Green diamond indicates the position of the native microalga. Number at nodes represents the percentages of bootstrap resampling based on 500 replicates. (**B**) Microalga morphologies at different light conditions, (a) autospores at constant light condition; (b), (c) and (d) cells in division with successive cleavages of protoplast at transition from constant light condition to darkness, (e) autospores at photoperiod condition and (f) vegetative cell at transition from photoperiod condition to darkness, single pyrenoid is indicated with arrow. All images were taken at 100x magnification. Bars represent 5 μm.

During the microalga isolation two different morphologies were observed, so the molecular identification was conducted by the amplification of partial ITS1-5.8S-ITS2 18S rRNA regions from each morphotype, resulting 100% identical between them and were identified as *Neochloris aquatica* after the Blast and multiple alignment analysis (Figs 1A and S1).

Looking for the best culture conditions for microalga axenic culture, different morphologies were detected according to the availability of light. In cultures exposed to constant light (Fig 1B, a) as well as photoperiod condition (Fig 1B, e), round-shaped autospores with a size range of 3–7 μm were observed. In transition from constant light to darkness, more than 27% of division cells (cleavage of protoplast) was found (Fig 1B, b-d), while in transition from photoperiod condition to darkness, cells of size range of 10–20 μm with a single pyrenoid were obtained (Fig 1B, f (arrow)), and no motile cells during darkness condition were detected. As the morphology found in field samples was similar to the one observed under continuous light, this morphotype was selected to perform further mosquito assays.

### Impact of microalga on oviposition substratum preferences by *Cx*. *quinquefasciatus* females

**Fig 2:**
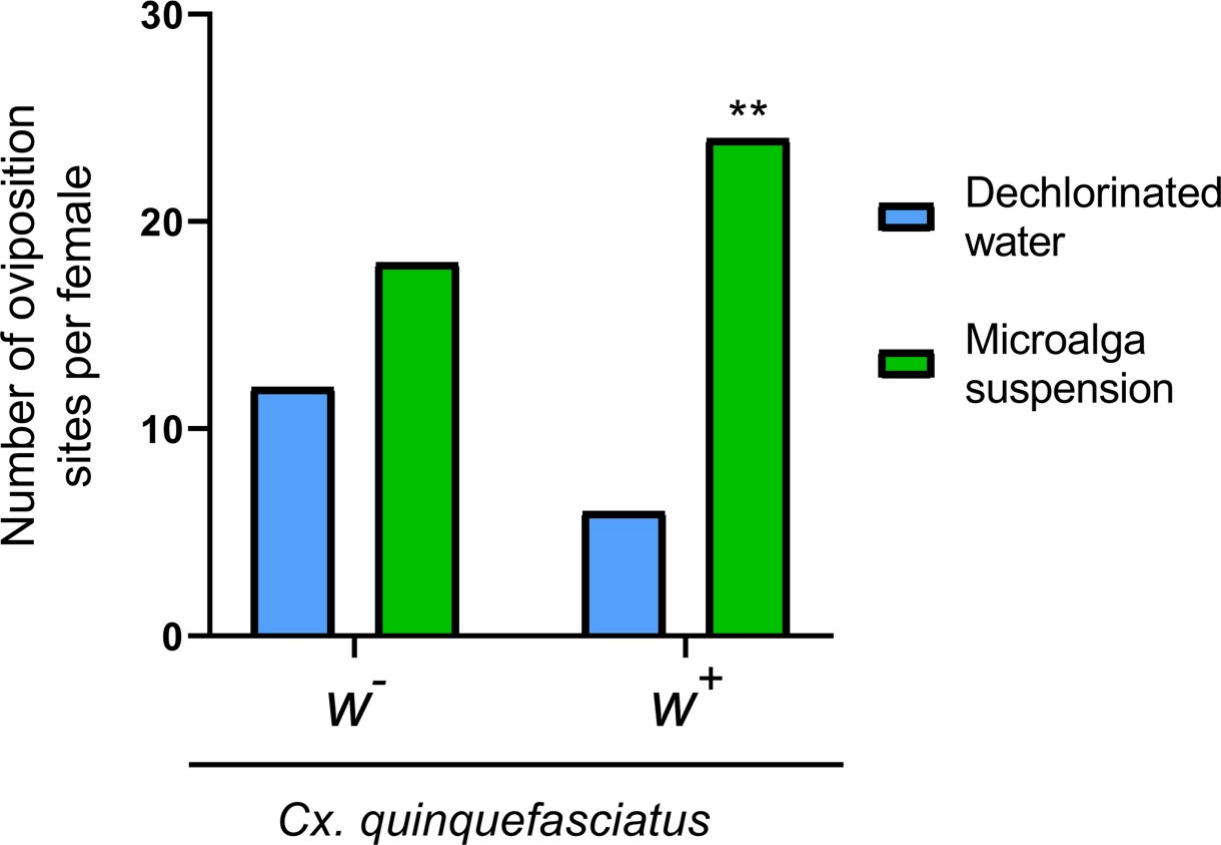
Selection of the oviposition site by gravid females of *Cx*. *quinquefasciatus* naturally infected with *Wolbachia* (*w*^*+*^) and *Wolbachia* free (*w*^*-*^) isolines in *N*. *aquatica* suspension or dechlorinated water. Female oviposition site selection was analyzed with binomial test (N = 30, *p* value = 0.05). ** *p* value < 0.001 indicate significant difference in oviposition site selection by gravid females from *w*^+^ isoline.

Gravid females of *Cx*. *quinquefasciatus*, naturally infected with *Wolbachia*, showed a statistically significant preference for the microalga suspension as oviposition substrate, so that 80% of them placed their egg rafts on the microalga suspension (binomial test: *p* < 0.001), while the 60% of the *w*^-^ females chose the microalga as their oviposition substrate, with no statistically significant differences between available substrates (binomial test: *p* = 0.3616) (Fig 2).

### Effect of microalga on *Cx*. *quinquefasciatus* neonate larvae

**Fig 3:**
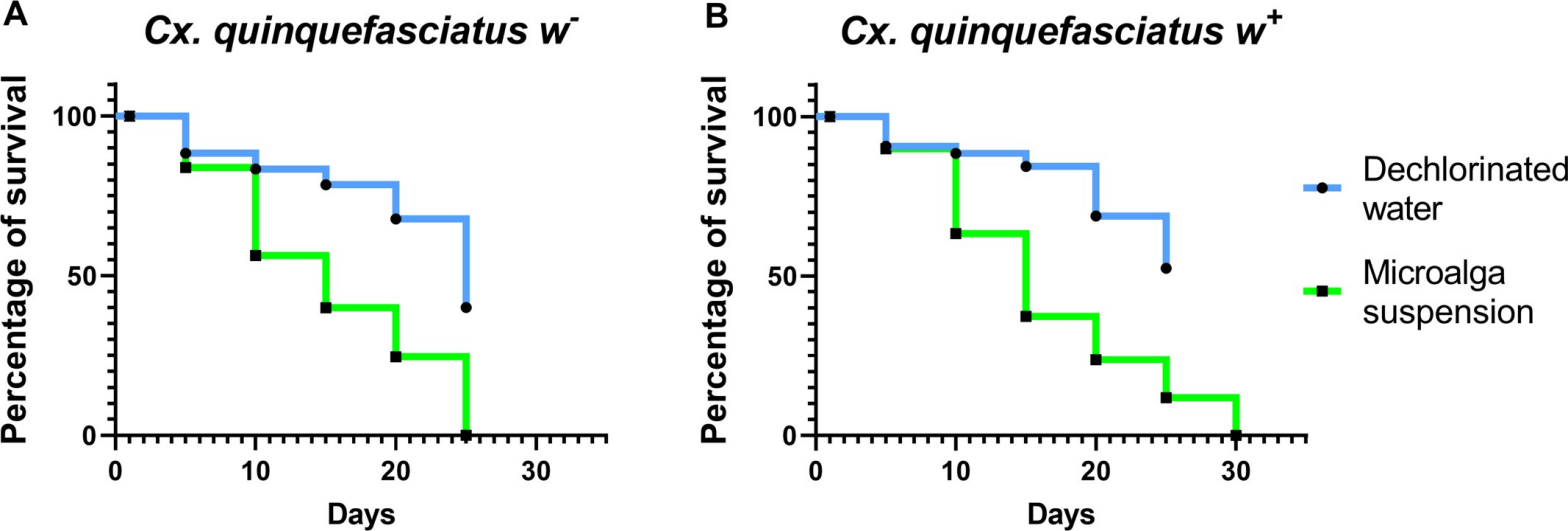
Survival curves of neonate *Cx*. *quinquefasciatus* larvae reared on *N*. *aquatica* suspension or dechlorinated water plus control diet (fish food). Kaplan-Meier survival curves per oviposition substrate; α = 0.05. (**A**) *Cx*. *quinquefasciatus w*^-^ and (**B**) *Cx*. *quinquefasciatus w*^+^.

To analyze the difference in survival between larvae developed in water or in algae, the Log-rank test (Mantel-Cox) was used, obtaining a *p* value < 0.0001. After 10 days of development, 45% of the larvae of *Cx*. *quinquefasciatus w*^-^ were dead, while for isoline *w*^+^ mortality was lower (37%). Although, after 25 days, all larvae from *w*^-^ isoline were dead, while a few *w*^+^ larvae (12%) were still alive (Fig 3).

### Effect of microalga on mosquito development and survival

In order to examine if the microalga was toxic against *Cx*. *quinquefasciatus*, second instar larvae of each mosquito line (*w*^*+*^ and *w*^*-*^) were fed with microalga suspension, as only food source compared with larvae reared in dechlorinated water and fed with fish food as a control.

**Fig 4:**
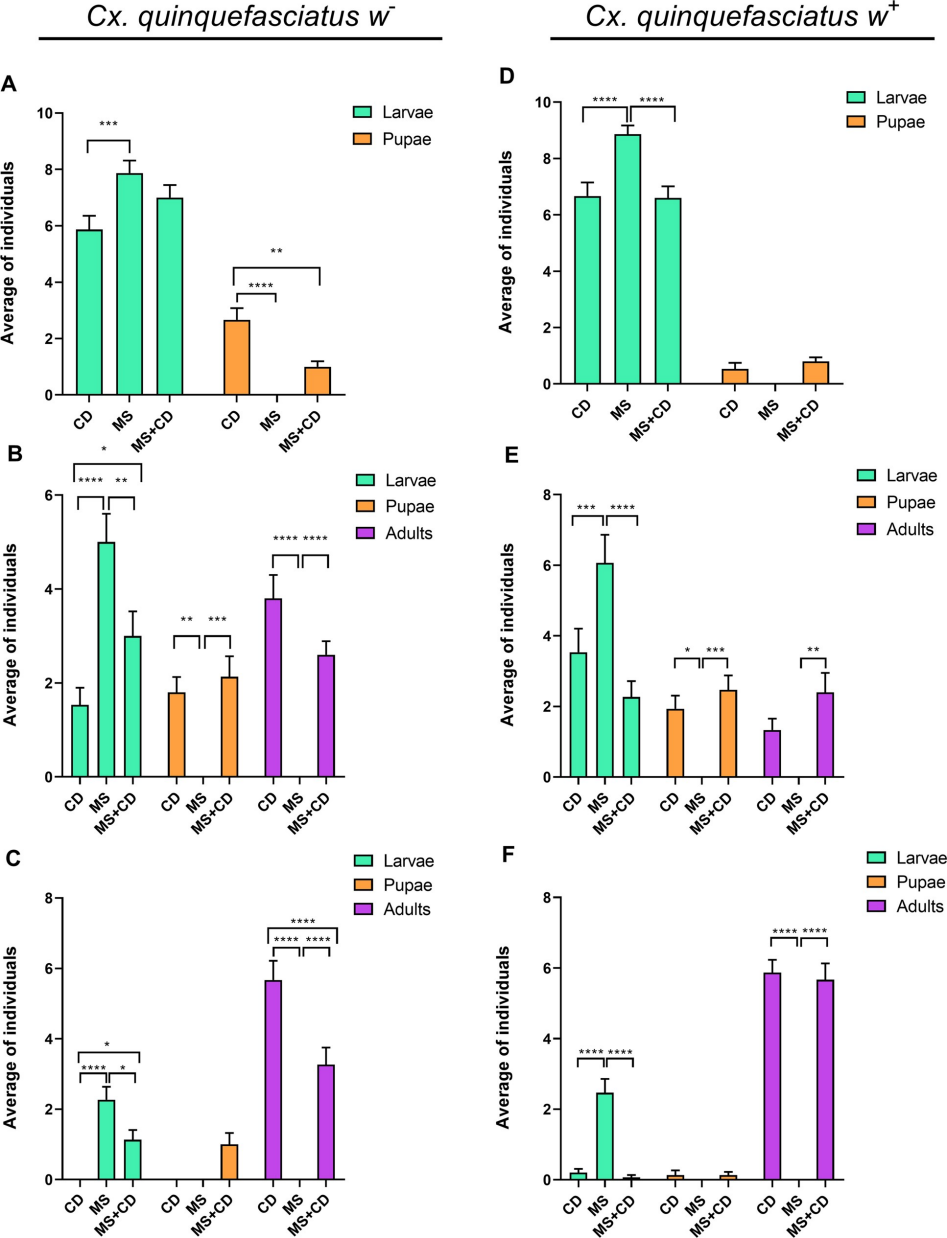
Development of *Cx*. *quinquefasciatus* lines fed on *N*. *aquatica* suspension (MS), microalga suspension plus control diet (MS+CD) and dechlorinated water with control diet (CD). Proportion of larval stages, pupa and adult among diet treatments for *Cx*. *quinquefasciatus w*^+^ and *w*^-^ at different times. (**A** and **D**) at 5 days, (**B** and **E**) at 10 days, (**C**) at 15 days and (**F**) at 20 days. Graphs represent mean ± SEM of 3 independent experiments of 5 replicates each, initial n = 50 per experiment, statistical differences between development stages among diets were determined using 2-Way ANOVA (* = *p* < 0.05, ** = *p* < 0.01, *** = *p* < 0.001, **** = *p* < 0.0001).

**Fig 5:**
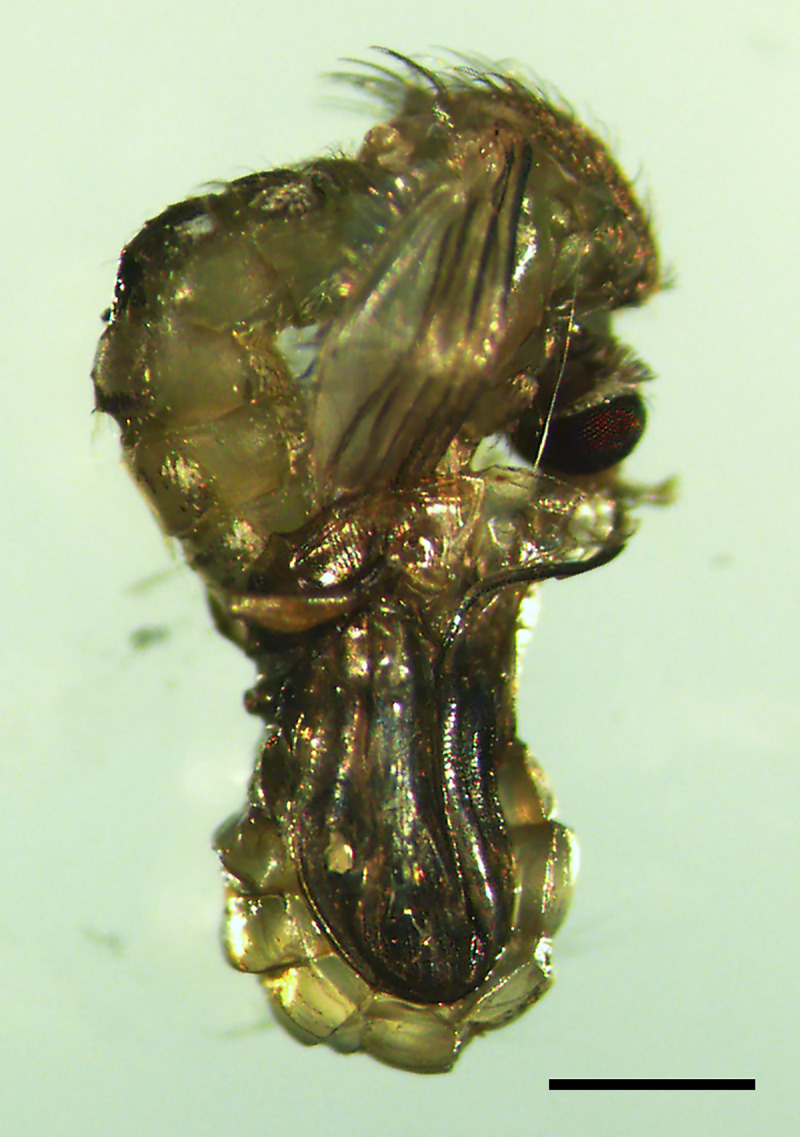
*Cx*. *quinquefasciatus w*^-^ reared on microalga suspension plus control diet. Some adults were unable to emerge from pupae. Bar indicates 5 mm.

Larvae reared on microalga suspension could not complete their life cycle, observing that their growth stopped at the second/third larval instar, and dying throughout the assay; at day 20, only 15% of the *w*^+^ larvae remained alive while all *w*^-^ larvae died (Fig 4). At the end of the assay, the isolines fed with control diet, completed their life cycle: *Wolbachia* free isoline within 15 days, while *Wolbachia* infected isoline presented higher survival rate, and by day 20 not all individuals reached adulthood. To analyze whether the observed effect was related to a nutritional requirement, larvae of both isolines were fed with suspension of microalga but supplemented with a control diet. In this case, larvae from both isolines were able to complete the life cycle reaching adulthood; however, for the *w*^-^ isoline some larvae and pupae were not able to molt to the next stage and about 10% of pupae aborted during the emergence to their adult form (Fig 5).

**Fig 6:**
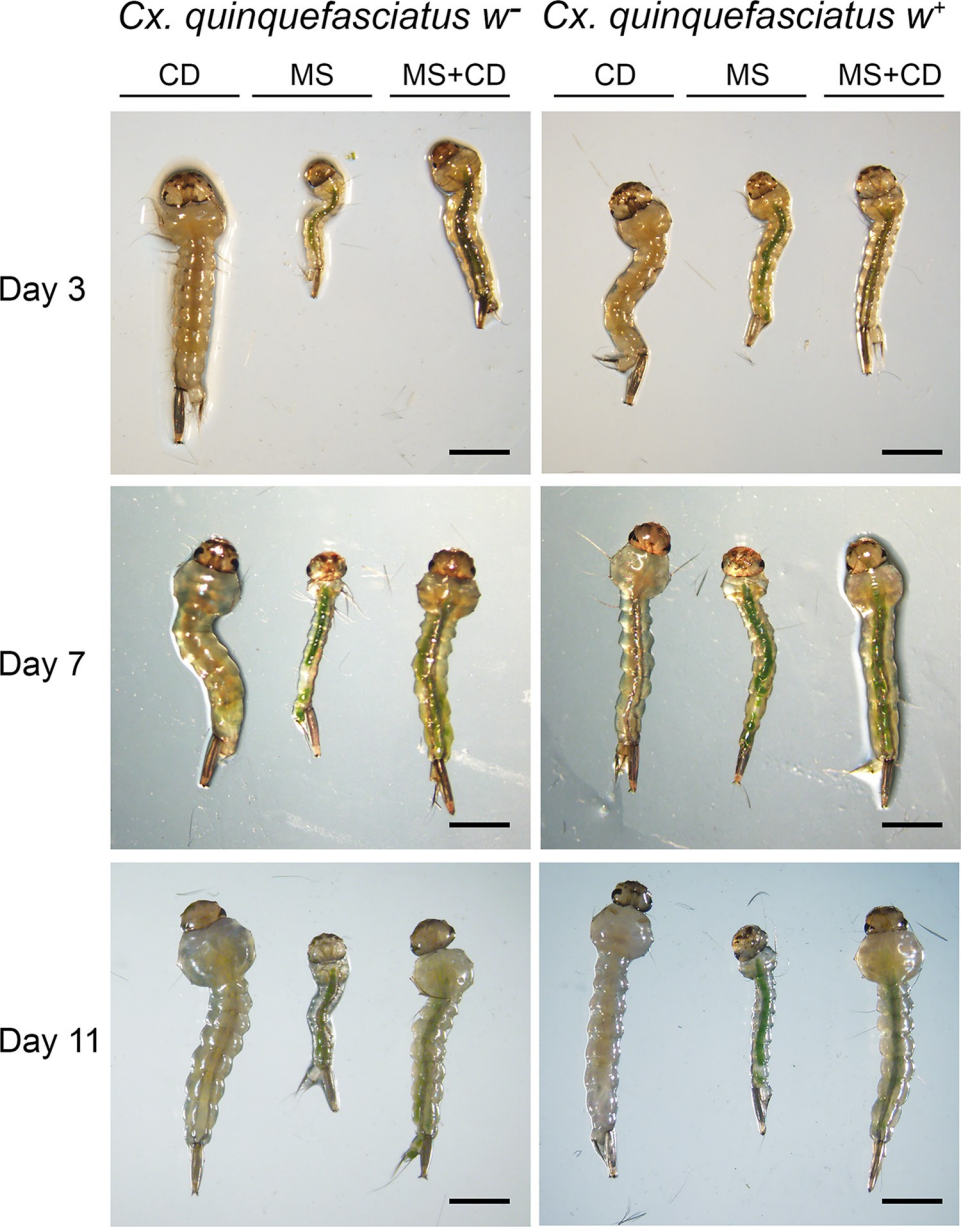
Total larvae length. *Cx*. *quinquefasciatus w*^-^ and *w*^+^ larvae fed on control diet (CD), microalga suspension (MS) or microalga suspension plus control diet (MS+CD) at days 3, 7 and 11. Bars indicate 1 mm.

To evaluate the effects of *N*. *aquatica* as nutrient source, total length of larvae was analyzed in the three feeding conditions at days 3, 7 and 11 (Fig 6).

To determine which of the variables explain the effect seen in length of *Cx*. *quinquefasciatus w*^+^ and *w*^-^ larvae, the results obtained were analyzed by GLMM model considering variables day, diet and isoline as explanatory variables (S2 Table). After that post-hoc comparisons were made using the Wilcoxon rank-sum test *p* values for total larval lengths (Table 1).

**Table 1:** Post-hoc pairwise Wilcoxon rank sum test *p* values for total larvae length, with comparisons between control diet (CD), microalga suspension (MS) and microalga suspension plus control diet (MS+CD) for *Cx*. *quinquefasciatus w*^+^ and *w*^*-*^ isolines.

			Isoline *w*^-^			Isoline *w*^-^			Isoline *w*^+^			Isoline *w*^+^		
			Day 3			Day 7			Day 3			Day 7		
			CD	MS	MS+CD	CD	MS	MS+CD	CD	MS	MS+CD	CD	MS	MS+CD
Isoline *w*^-^	Day 3	CD	-	-	-	-	-	-	-	-	-	-	-	-
		MS	3.9e-10	-	-	-	-	-	-	-	-	-	-	-
		MS+CD	1.0000	3.3e-07	-	-	-	-	-	-	-	-	-	-
Isoline *w*^-^	Day 7	CD	<2e-16	<2e-16	<2e-16	-	-	-	-	-	-	-	-	-
		MS	0.5613	1.2e-09	0.0761	<2e-16	-	-	-	-	-	-	-	-
		MS+CD	<2e-16	<2e-16	<2e-16	0.1257	<2e-16	-	-	-	-	-	-	-
Isoline *w*^+^	Day 3	CD	0.4806	7.0e-14	0.0714	<2e-16	1.0000	<2e-16	-	-	-	-	-	-
		MS	2.6e-07	1.0000	0.0002	<2e-16	2.0e-07	<2e-16	4.4e-11	-	-	-	-	-
		MS+CD	1.3e-09	<2e-16	1.1e-11	2.2e-15	0.0018	<2e-16	0.0002	<2e-16	-	-	-	-
Isoline *w*^+^	Day 7	CD	1.8e-14	<2e-16	<2e-16	1.0000	7.1e-12	0.0761	1.5e-11	<2e-16	9.6e-08	-	-	-
		MS	9.7e-16	<2e-16	<2e-16	5.2e-11	1.9e-09	1.4e-15	3.5e-09	<2e-16	0.1131	0.0003	-	-
		MS+CD	<2e-16	<2e-16	<2e-16	6.5e-06	<2e-16	0.1131	<2e-16	<2e-16	<2e-16	5.7e-05	<2e-16	-

On day 3, larvae of *Cx*. *quinquefasciatus w*^-^ isoline fed with microalga suspension (MS) were significantly shorter than larvae fed with control diet, a similar, but much less drastic effect was observed, when larvae were fed with microalga suspension supplemented with control diet. The size differences between the larvae fed with microalga or with microalga plus control diet were much smaller in the naturally infected *Wolbachia* isoline.

**Fig 7:**
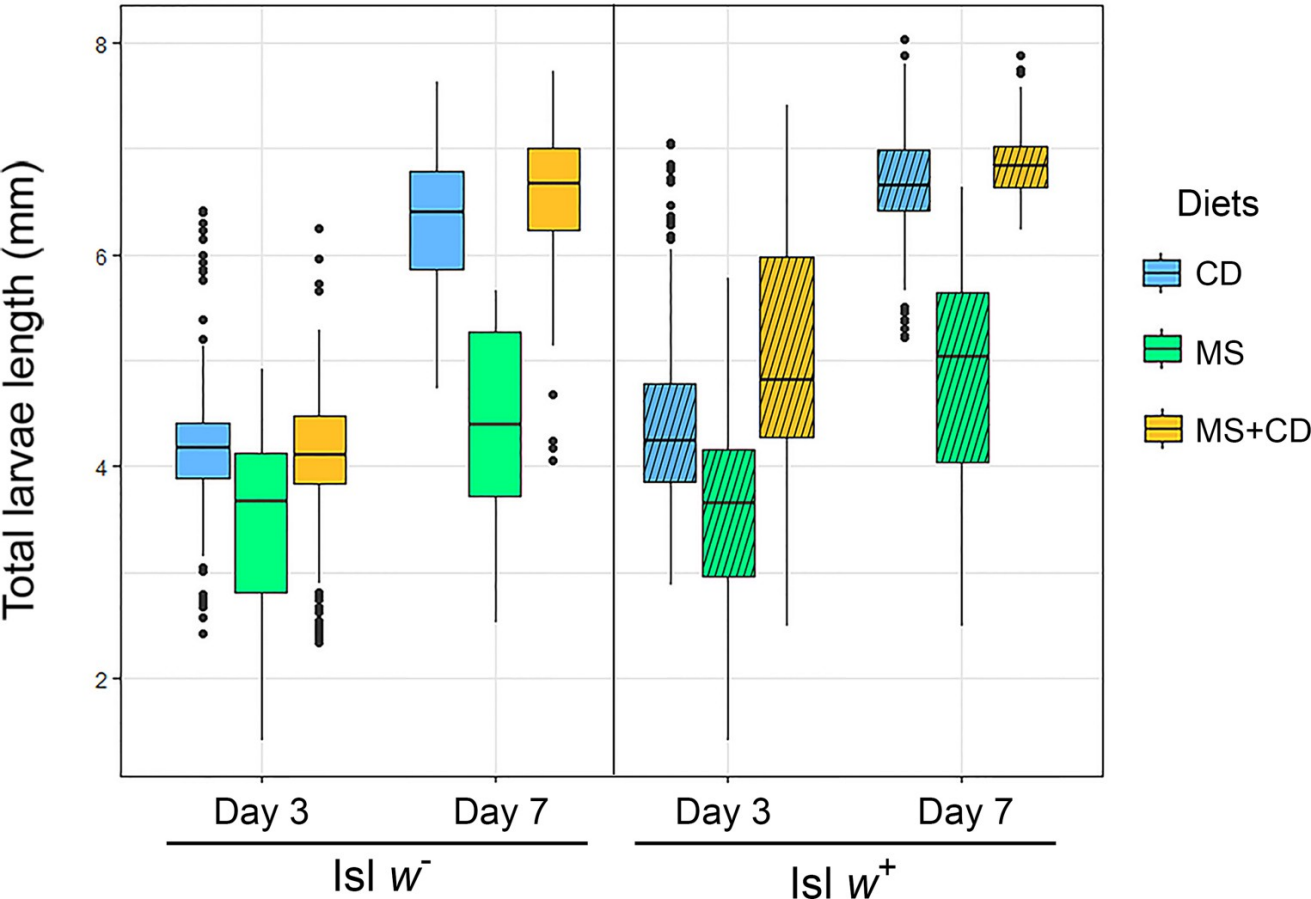
Effect of variables day, diet and isoline on total larvae length. Larval lengths were measured at days 3 and 7, among diets: control diet (CD), microalga suspension (MS) and microalga suspension plus control diet (MS+CD) respectively; for both isolines (Isl) corresponding to *w*^*-*^ (solid color) and *w*^*+*^ (diagonal pattern).

On the other hand, double interaction between day 7 and MS diet, affected differently depending on the presence or absence of *Wolbachia*; at day 7, *w*^-^ larvae fed only with microalga did not increase significantly in length compared to day 3; however, their size did increase when the microalga diet was supplemented with control diet. Regarding the *w*^+^ line, on day 7, larvae fed only with microalga showed a greater length compared to the larvae of the *Wolbachia* free mosquito line under the same experimental conditions (S2 Table and Fig 7). Data from day 11 were not included due to most of the larvae of both isolines fed on microalga had died, and the larvae fed with a control diet or alga supplemented with control diet, had already pupated.

According to Akaike Information Criterion (AIC), the top-ranking model (*w*_*i*_ = 0.999) was the only competitive one and had more support than the second-ranking model (ΔAIC > 4) (S3 Table).

**Fig 8:**
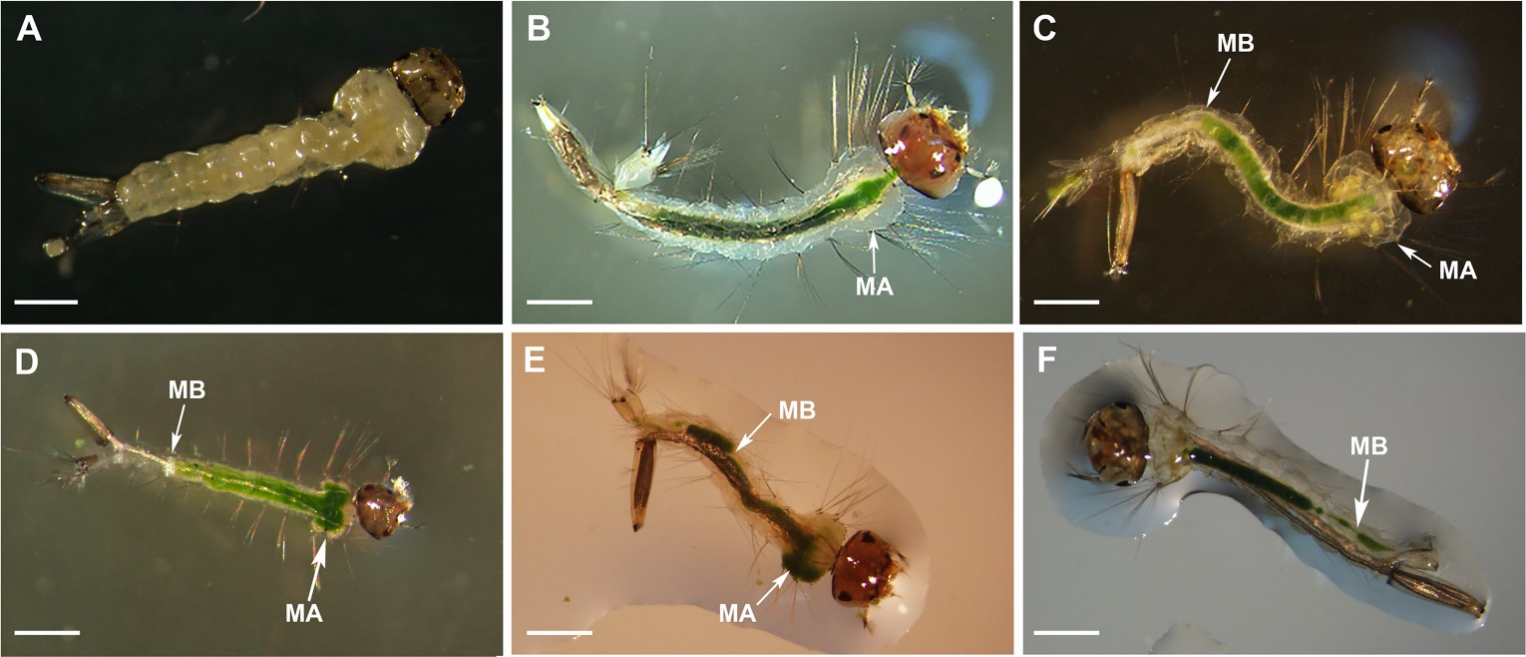
Morphological alterations of *Cx*. *quinquefasciatus w*^-^ larvae fed on *N*. *aquatica* suspension under stereomicroscope observation. Morphological alterations (MA) and midgut blockage (MB). (**A**) Larvae fed with control diet after 11 assay days, bar indicates 1mm. (**B-F**) Larvae fed with microalga suspension after 11 days. Bars indicate 0.5 mm.

Drastic effects on *Cx*. *quinquefasciatus* larvae *w*^-^ fed with microalga suspension were observed (Fig 8), most of them showed abdominal and thoracic alterations, disruptions in the gut and a decreased fat body (Fig 8B–8F). Also, alterations of the gut continuity and the blockage by food bolus were observed (Fig 8C–8F). Larvae fed with control diet showed thorax and abdominal segments with a normal appearance (Fig 8A).

### Midgut extract activity on the cellular integrity of *N*. *aquatica*

**Fig 9:**
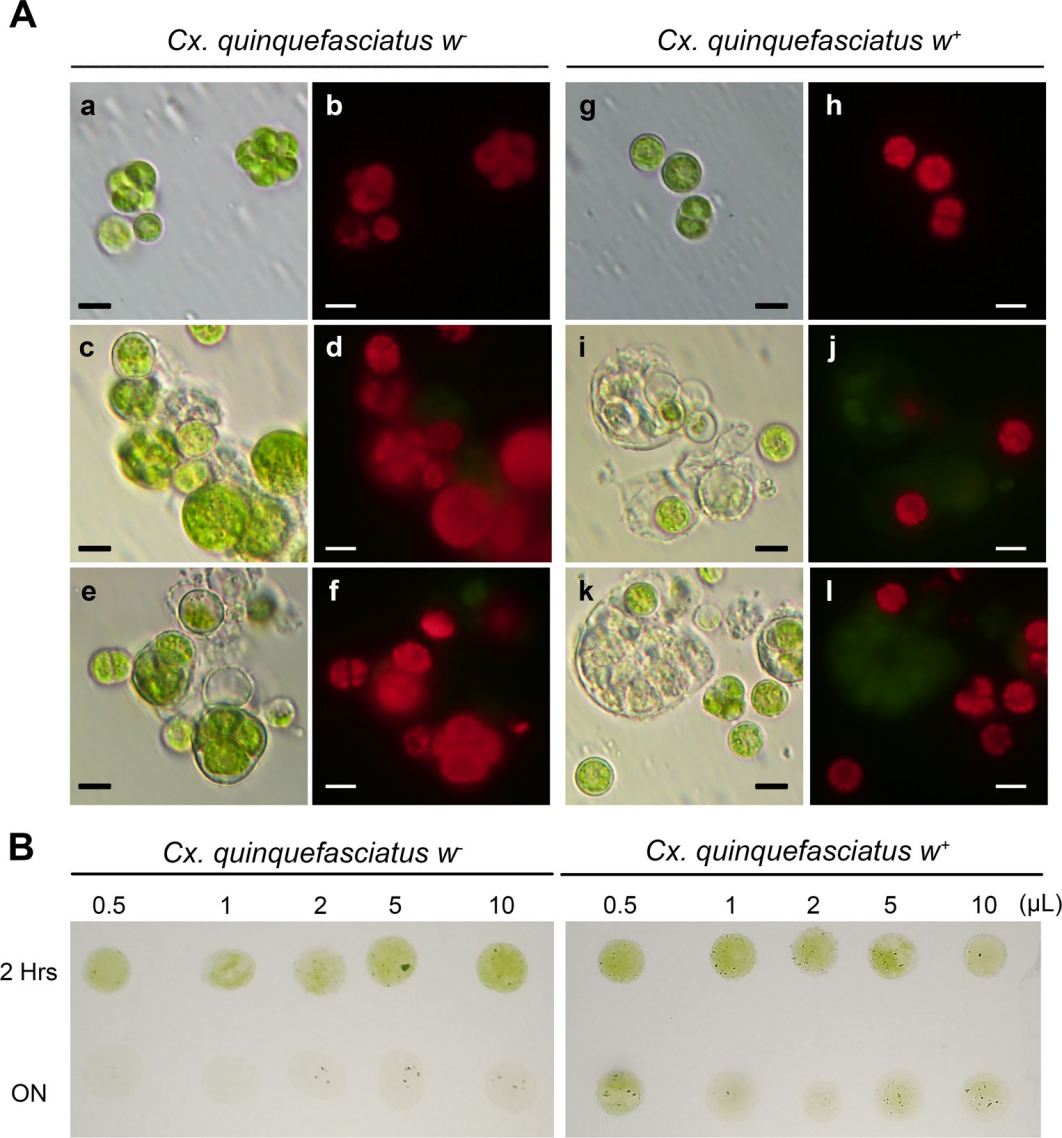
**(A) Larval midgut extracts of *Cx*. *quinquefasciatus* isolines *w***^**-**^ **and *w***^**+**^ **activity against *N*. *aquatica* cells.** (**a**, **c**, **e**, **g**, **i** and **k**) bright field microscopy shows cells integrity, (**b**, **d**, **f**, **h**, **j** and **l**) fluorescence field microscopy shows chlorophyll autofluorescence. (**a**-**b** and **g-h**) microalga culture without midgut extract. Mixture of 10 μL of microalga plus 5 μL (**c**-**d** and **i-j**) or 10 μL of midguts extract (**e-f** and **k-l**). Bars represent 5 μm. Incubations were carried out at 37°C for 2 hours. **(B)** Different volumes of midgut extract (0.5, 1, 2, 5 and 10 μL) and microalga culture (10 μL) were mixed and incubated at 37°C for 2 hours or overnight, and then sown and incubated in BG-11 agar plates in controlled conditions of light and temperature, for 24 hours.

Qualitative results showed that incubation of midgut extracts with *N*. *aquatica* cells had a negative effect on the viability and growth of alga cells (Fig 9). Broken cell walls without cellular content were observed and they increased as a function of the volume of midgut extract and the incubation time. In damaged cells, the fluorescence of chlorophyll was not detected, and in all evaluated conditions, resistant forms were observed. The results of Fig 9 show that the number of cells capable of growing and recovering after incubation with the midgut extract decreases as a function of time and incubation volume.

## Discussion

The goal of this work was to study a natural sample of a microalga found in an artificial breeding site where there were no immature stages of mosquitoes, even though a large number of larvae and pupae were found in the nearby hatcheries. The microalga, isolated from rainwater accumulated in a discarded tire, was determined as *N*. *aquatica*, one of the 17 species of the *Neochloris* genus identified up to date [38]. This genus, firstly characterized by Starr in 1955 [39,40] as a member of *Chlorococcaceae* family, it is described as vegetative cells with a parietal chloroplast with at least one pyrenoid in it [41,42]. While searching for optimal culture conditions, we obtained autospores in *N*. *aquatica* cultures exposed to constant light, as well as in photoperiod conditions, where division cells and differences in size were observed in transitions of constant light to darkness, and photoperiod to darkness, respectively. Detected morphologies are also seen in other *Chlorophyceae* [43], and as previously described by Přibyl [29], no motile cells were found in dark conditions due to their asexual reproduction by autospores.

Many microalga metabolites have been described so far, most of them allelochemicals [13,14], which give them relevant advantages. These compounds have effects on reproduction, survival and in inhibiting the growth of competitive species, and maybe this is the reason why the initial culture, in the mosquito breeding site, was almost a monoalgal culture. On the other hand, microalgae produce volatiles compounds that might act as attractants for some insects, including mosquitoes, such as fatty acids derivatives [44,45], terpenoids [46], alcohols [47], and aldehydes [48]. Mosquitoes depend on olfactory signals to carry out behavioral activities, such as locating preys or hosts, selecting places to lay their eggs, courtship, and others, that they detect through their sensory organs. The main olfactory stimuli for females are carbon dioxide, lactic and caproic acids, octenol, acetone, butanone, and phenolic compounds. Some of these are used for choosing potential larval habitats in order to maximize the fitness of their offspring [49]. It is expected that gravid females choose to lay their eggs in habitats that offer the highest probability for their offspring to reach adulthood, therefore, one of the questions is if the alga releases some repellent compound that inhibits the oviposition. However, in this work, *Cx*. *quinquefasciatus* adult females, from both populations (*w*^+^ and *w*^-^), showed a marked preference for *N*. *aquatica* suspension as oviposition substrate, mainly the *w*^+^ isoline, and the choice of the oviposition site could be due to the higher concentration of organic matter in the containers with alga. In insects such as *Drosophila melanogaster*, the presence of the endosymbiotic bacterium *Wolbachia* may improve the response to olfactory signals [50,51]; but, further studies to elucidate the role of *Wolbachia* in the selection for oviposition of *Cx*. *quinquefasciatus*, are needed.

Here we described a negative effect of *N*. *aquatica* on the *Cx*. *quinquefasciatus* larvae populations for the first time. We detected that approximately half of the neonate larvae of both populations (*w*^+^ and *w*^-^) that were reared in the microalga suspension, died after 10 to 15 days (Fig 3), showing a decreased fat body (Fig 8). 20 days later only a few *w*^+^ larvae remained alive at L2 stage (Figs 3 and 4). Other studies show that *Ae*. *aegypti* larvae fed on *Chlorella* spp. were able to complete their development to adulthood [52], but in this case *N*. *aquatica* kills mosquito larvae maybe because it could be indigestible as it is proposed by Díaz-Nieto *et al*. [30], who detected that *Cx*. *pipiens* larvae were unable to develop beyond L3 larvae when fed on *Chlorella sorokiniana*. *Cx*. *quinquefasciatus* larvae are non-selective feeding filters of components of the aquatic microflora including bacteria, protozoa, fungi and microalgae [14,52–54]. In particular, microalgae are significant part of mosquitoes nutritional source, providing in general, an adequate alimentary source; however, some of them can be indigestible [55] causing nutritional deprivation with negative effects on the larvae growth and the adult body size in *Culex*, *Aedes* or *Anopheles* species.

In this work, larval development was monitored considering the length of the larvae as a parameter of growth [56]. A negative effect was observed on the total length when *N*. *aquatica* suspension was used as the unique nutrient source (MS in Fig 6). The length was similar between larvae fed on control diet (CD) and when were fed on the microalga supplemented with the control diet (MS+CD vs CD in Fig 6). Although, the presence of *Wolbachia* in larvae can bring a transitory advantage since the *w*^*+*^ larvae were longer (panel A, day 7 MS *w*^*-*^ vs panel B, day 7 MS *w*^*+*^ in Fig 7), both isolines (*w*^*+*^ and *w*^*-*^) fed with *N*. *aquatica* suspension, as the only source of nutrients, died before reaching the next developmental stage. According to previous reports, several species of microalgae, as *Chlorella*, *Scenedesmus*, *Pediastrum*, *Coelastrum* have cell walls indigestible for larvae; because the major compound of the cell wall is the sporopollenin, that it is not degraded by the digestive enzymes and also is resistant to chemical hydrolysis [14,53,57]. However, the composition of the cell wall of microalgae is variable and complex, even among taxonomically close species and can be composed of cellulose in combination with other complex sugars such as galactose, xylose, rhamnose, mannose or arabinose forming a rigid barrier and it can also contain sporopollenin [57,58]. Although, the composition of *N*. *aquatica* cell wall is unknown, another nearby microalga such as *Ettlia oleoabundans* (previously named *Neochloris oleoabundans*) has a cell wall composed by cellulose and proteins. This conclusion is reached after trying different enzymatic treatments to achieve cell disruption [58,59], but in *N*. *aquatica* these compounds could be combined with other sugars that make the microalgae indigestible for the larvae.

On the other hand, it has been reported that some microalgae have lethal effects or release toxic compounds in the aquatic environment [53]; among them, strains of *Oscillatoria agardhii*, which in addition to being hepatotoxic, can produce toxins that cause lesions in the epithelial cells of the midgut of *Ae*. *aegypti* larvae [60]. Here it is shown that, despite the fact that some larvae fed with *N*. *aquatica* supplemented with balanced fish food, achieved a similar larval size, the number of surviving mosquitoes was lower. Moreover, the accumulation of alga in the gut caused serious damage to its integrity. As seen in Fig 8B–8F, larval gut presented deformations occupying the thoracic area (Fig 8D–8E) and, in other cases, blockages and alterations were observed when compared with control growing in optimal conditions (Fig 8A). Besides, we observed rupture of the *N*. *aquatica* cell walls, with increasing damage as the volume of larval midgut extract was added and the incubation time increased. This result strongly suggests lytic activity of the larval extract that could lead to the release of alga components that could have a toxic effect on the growth and development of mosquito larvae (Fig 9), indicating that at least some *N*. *aquatica* forms, could produce bioactive compounds [13,14,53]. Mosquito larvae that ingest alga cells do not achieve normal development, and this damage is not completely reversed when supplementing the diet with a diet rich in nutrients, confirming that the effect produced is not due to a poor diet. The bioactive compounds generated by *N*. *aquatica* could be applied as components that could be used in innovative biological control products for the control of mosquito populations in integrated vector management programs.

Finally, in *w*^-^ isoline, some pupae were unable to complete their metamorphosis to an adult stage, confirming again that carrying *Wolbachia* confers benefits on the mosquito development. We have previously reported that between *w*^-^ and *w*^+^ *Cx*. *quinquefasciatus* isolines fecundity (eggs laid per female) and fertility (total eggs hatched) rates show no significant differences, but the *w*^+^ line presented higher survival rates than *w*^-^ line and is less susceptible to the effect of entomopathogenic bacteria [28]. Likewise, it has also been shown that the *Wolbachia* amount is higher in *Cx*. *pipiens* lines resistant to organophosphorus insecticides than in susceptible mosquitoes [61]. These effects may be due to a response to the increase in gene expression related to the immune system induced under the presence of the endosymbiont bacterium, and due to the *Wolbachia* capability to provide key metabolites in its nutritional mutualistic relationship [62].

## Supporting information

S1 Fig Multiple sequence alignment of ITS1-5.8s-IT2 DNA fragment from native microalga and sequences obtained using BLAST algorithm. Multiple sequence alignment was constructed using ClustalW algorithm considering a IUB (DNA Weight matrix), identical or similar residues are shaded in black background using Box Shade.
(DOCX)

S1 Table Antibiotic sensitivity against native isolate of microalga and accompanying bacterium. (DOCX)

S2 Table Generalized linear mixed model results for total larvae length. Variable diet included three levels (CD: control diet, MS: microalga suspension and MS+CD: microalga suspension plus control diet), day included two levels (day 3 and day 7) and isoline included two levels (*w*^-^ isoline and *w*^+^ isoline). The factors and interactions highlighted in boldface type were found to have significant effects.
(DOCX)

S3 Table Statistical models ranked by Akaike’s Information Criterion. (DOCX)
