# Supplementary material for: Culex quinquefasciatus larvae development arrested when fed on Neochloris aquatica
Source: PLoS Negl Trop Dis. 2021 Dec 3;15(12):e0009988. doi: 10.1371/journal.pntd.0009988 (PMC8641890; doi:10.1371/journal.pntd.0009988)
Supplement: S1 Fig — Multiple sequence alignment was constructed using ClustalW algorithm considering a IUB (DNA Weight matrix), identical or similar residues are shaded in black background using Box Shade. (DOCX) [file pntd.0009988.s001.docx]

**S1 Fig**


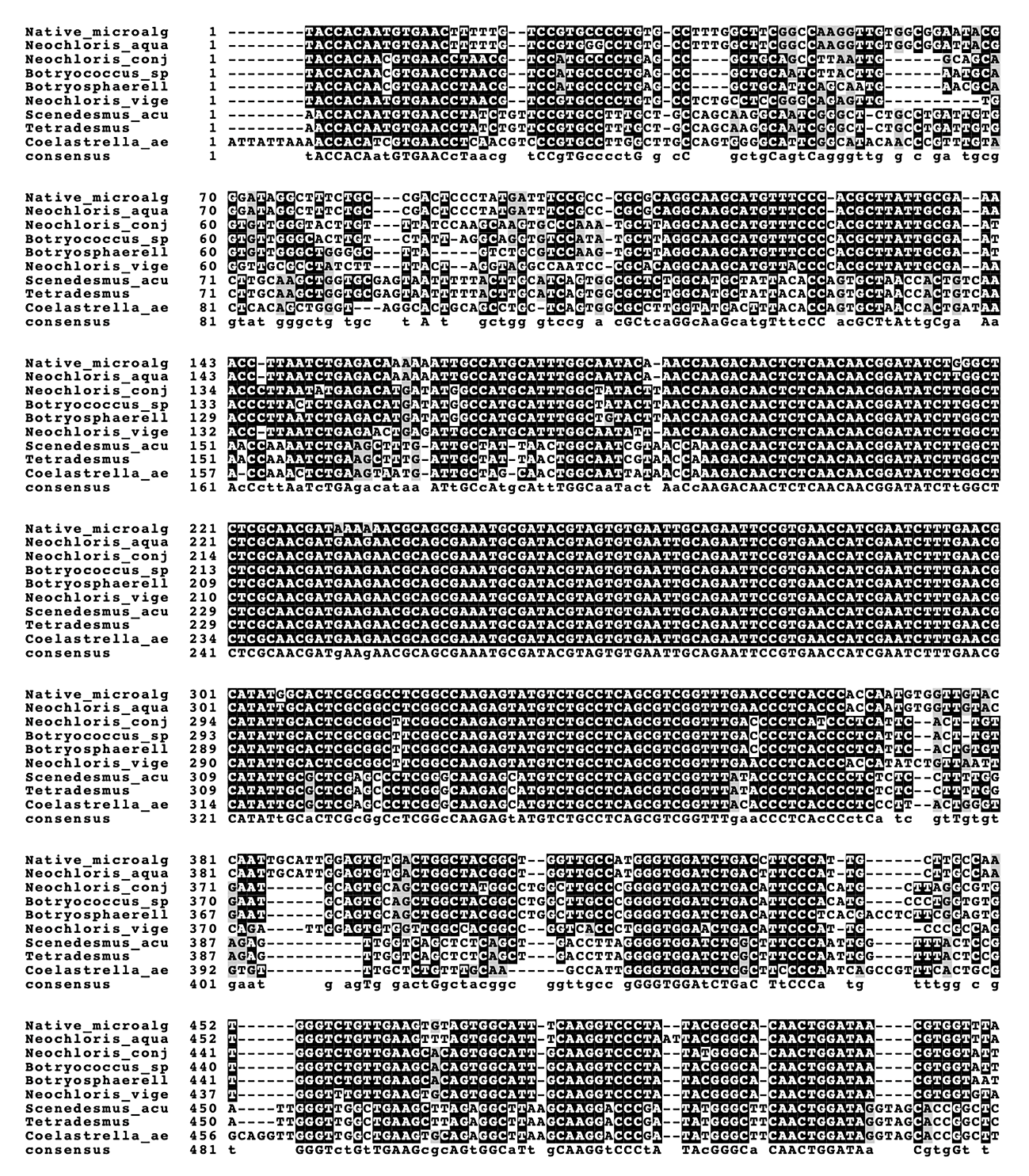


**S1 Fig.** Multiple sequence alignment of ITS1-5.8S-ITS2 DNA fragment from native microalga and sequences obtained using BLAST algorithm. Multiple sequence alignment was constructed using ClustalW algorithm considering a IUB (DNA Weight matrix), identical or similar residues are shaded in black background using Box Shade.
