# Supplementary material for: Culex quinquefasciatus larvae development arrested when fed on Neochloris aquatica
Source: PLoS Negl Trop Dis. 2021 Dec 3;15(12):e0009988. doi: 10.1371/journal.pntd.0009988 (PMC8641890; doi:10.1371/journal.pntd.0009988)
Supplement: S1 Table — (DOCX) [file pntd.0009988.s002.docx]

**S1 Table**

**S1 Table. Antibiotic sensitivity against native isolate of microalga and accompanying bacterium.**

| **Antibiotics** | **Antibiogram concentration**  **(µg/mL)** | **Bacterium* sensitivity** | **Microalga* sensitivity** |
| --- | --- | --- | --- |
| Ampicillin | 50 | - | N/A |
| Carbenicillin | 5 | - | N/A |
| Carbenicillin | 100 | ++ | ++ |
| Chloramphenicol | 30 | - | N/A |
| Erythromycin | 30 | - | N/A |
| Gentamicin | 50 | +++ | ++ |
| Kanamycin | 30 | +++ | ++ |
| Nalidixic acid | 30 | + | N/A |
| Neomycin** | 30 | ++ | + |
| Rifampicin | 30 | + | N/A |
| Spectinomycin | 50 | - | N/A |
| Streptomycin | 50 | + | N/A |
| Tetracycline | 30 | +++ | ++ |

*Degree of sensitivity: + weak sensitivity, ++ moderate sensitivity and +++ high sensitivity. N/A: not available. ** Selected antibiotic. Antibiotic selection is made in BG11 agar culture medium.
