# Supplementary material for: Culex quinquefasciatus larvae development arrested when fed on Neochloris aquatica
Source: PLoS Negl Trop Dis. 2021 Dec 3;15(12):e0009988. doi: 10.1371/journal.pntd.0009988 (PMC8641890; doi:10.1371/journal.pntd.0009988)
Supplement: S2 Table — Variable diet included three levels (CD: control diet, MS: microalga suspension and MS+CD: microalga suspension plus control diet), day included two levels (day 3 and day 7) and isoline included two levels (w- isoline and w+ isoline). The factors and interactions highlighted in boldface type were found to have significant effects. (DOCX) [file pntd.0009988.s003.docx]

**S2 Table**

**S2 Table.** **Generalized linear mixed model results for total larvae length.** Variable diet included three levels (CD: control diet, MS: microalga suspension and MS+CD: microalga suspension plus control diet), day included two levels (day 3 and day 7) and isoline included two levels (*w*^-^ isoline and *w*^+^ isoline). The factors and interactions highlighted in boldface type were found to have significant effects.

|  | **Estimate** | **Std. Error** | **t value** | **Pr (>\|z\|)** | **Estimate** |
| --- | --- | --- | --- | --- | --- |
| (Intercept) = Day 3 + Isoline *w*^-^ + CD | 0.237272 | 0.004052 | 58.561 | **< 2e-16** | *** |
| MS | 0.048727 | 0.006183 | 7.881 | **3.26e-15** | *** |
| MS+CD | 0.010070 | 0.005651 | 1.782 | **0.07476** | . |
| Day 7 | -0.078356 | 0.005434 | -14.419 | **< 2e-16** | *** |
| Isoline *w*^+^ | -0.014964 | 0.005318 | -2.814 | **0.00489** | ** |
| MS: Day 7 | 0.020622 | 0.008574 | 2.405 | **0.01617** | * |
| MS+CD: Day 7 | -0.014992 | 0.007477 | -2.005 | **0.04497** | * |
| MS: Isoline *w*^+^ | 0.004361 | 0.008473 | 0.515 | 0.60682 |  |
| MS+CD: Isoline *w*^+^ | -0.030477 | 0.007455 | -4.088 | **4.35e-05** | *** |
| Day 7: Isoline *w*^+^ | 0.005955 | 0.007205 | 0.827 | 0.40852 |  |
| MS: Day 7: Isoline *w*^+^ | -0.015930 | 0.011403 | -1.397 | 0.16243 |  |

*** (0), ** (0.001), * (0.01), . (0.05), (0.1) significance codes.
