# Supplementary material for: Culex quinquefasciatus larvae development arrested when fed on Neochloris aquatica
Source: PLoS Negl Trop Dis. 2021 Dec 3;15(12):e0009988. doi: 10.1371/journal.pntd.0009988 (PMC8641890; doi:10.1371/journal.pntd.0009988)
Supplement: S3 Table — (DOCX) [file pntd.0009988.s004.docx]

**S3 Table**

**S3 Table. Statistical models ranked by Akaike’s Information Criterion.**

| **Model** | ***k*** | **log-Likelihood** | **AIC** | **ΔAIC** | ***w_i_*** |
| --- | --- | --- | --- | --- | --- |
| Day*Isl*Dt | 14 | -1785,595 | 3599,2 | 0.00 | **0.999** |
| (Day:Isl:Dt) + Day + Isl + Dt | 12 | -1795,364 | 3614,7 | 15.54 | 0.000 |
| (Day:Isl) + (Isl: Dt) + Day + Isl + Dt | 10 | -1797,650 | 3615,3 | 16.11 | 0.000 |
| (Day:Isl) + (Day: Dt) + Day + Isl + Dt | 10 | -1798,905 | 3617,8 | 18.62 | 0.000 |
| (Day:Isl) + Day + Isl + Dt | 8 | -1801,109 | 3618,2 | 19.03 | 0.000 |
| (Day: Dt) + (Isl: Dt) + Day + Isl + Dt | 11 | -1800,509 | 3623,0 | 23.83 | 0.000 |
| (Isl: Dt) + Day + Isl + Dt | 9 | -1802,844 | 3623,7 | 24.50 | 0.000 |
| Day + Isl + Dt | 7 | -1806,310 | 3626,6 | 27.43 | 0.000 |
| Day | 4 | -2080,351 | 4168,7 | 569.51 | 0.000 |
| Dt | 5 | -2211,166 | 4432,3 | 833.14 | 0.000 |
| Isl | 4 | -2324,173 | 4656,3 | 1057.16 | 0.000 |
| Null | 3 | -2337,548 | 4681,1 | 1081.91 | 0.000 |

Models with triple and double interactions as well as individual effects of variables day, isoline (Isl) and diet (Dt) were analyzed. *K*: number of parameters, AIC: Akaike’s Information Criterion, ΔAIC: differences of AIC scores between each model and the lowest-AIC model, *w_i_*_:_ model Akaike weight. The only competitive model is in bold typeface.
